# Supplementary material for: Multi‐dataset identification of innovative feature genes and molecular mechanisms in keratoconus
Source: J Cell Mol Med. 2024 Sep 19;28(18):e70079. doi: 10.1111/jcmm.70079 (PMC11412914; doi:10.1111/jcmm.70079)
Supplement: Supplementary file 2 — Material S2. [file JCMM-28-e70079-s002.zip › 7.GSEA analysis/1.ARL11/KEGG_GLYCOLYSIS_GLUCONEOGENESIS.html]

Details for gene set KEGG\_GLYCOLYSIS\_GLUCONEOGENESIS[GSEA]

|  || Dataset | ARL11.group.cls#h\_versus\_l |
| Phenotype | group.cls#h\_versus\_l |
| Upregulated in class | l |
| GeneSet | KEGG\_GLYCOLYSIS\_GLUCONEOGENESIS |
| Enrichment Score (ES) | -0.27831107 |
| Normalized Enrichment Score (NES) | -1.0193133 |
| Nominal p-value | 0.42771083 |
| FDR q-value | 1.0 |
| FWER p-Value | 0.999 |
Table: GSEA Results Summary

  

Fig 1: Enrichment plot: KEGG\_GLYCOLYSIS\_GLUCONEOGENESIS      
 Profile of the Running ES Score & Positions of GeneSet Members on the Rank Ordered List

  

| SYMBOL | TITLE | RANK IN GENE LIST | RANK METRIC SCORE | RUNNING ES | CORE ENRICHMENT || 1 | HK3 | na | 938 | 0.397 | 0.0467 | No |
| 2 | ADH4 | na | 3617 | 0.215 | 0.0093 | No |
| 3 | FBP2 | na | 4838 | 0.171 | 0.0059 | No |
| 4 | PCK1 | na | 5840 | 0.141 | 0.0032 | No |
| 5 | ADH6 | na | 6053 | 0.136 | 0.0223 | No |
| 6 | LDHAL6A | na | 6578 | 0.122 | 0.0299 | No |
| 7 | ACSS1 | na | 6583 | 0.122 | 0.0524 | No |
| 8 | DLD | na | 9166 | 0.072 | -0.0086 | No |
| 9 | ENO2 | na | 9323 | 0.070 | -0.0001 | No |
| 10 | PFKP | na | 9473 | 0.068 | 0.0082 | No |
| 11 | ENO3 | na | 10243 | 0.057 | -0.0034 | No |
| 12 | AKR1A1 | na | 10312 | 0.056 | 0.0051 | No |
| 13 | ALDH7A1 | na | 10913 | 0.048 | -0.0033 | No |
| 14 | ALDH3B1 | na | 11840 | 0.038 | -0.0230 | No |
| 15 | FBP1 | na | 12362 | 0.033 | -0.0319 | No |
| 16 | PDHA2 | na | 12632 | 0.030 | -0.0341 | No |
| 17 | ALDH3A2 | na | 13283 | 0.025 | -0.0482 | No |
| 18 | LDHC | na | 13378 | 0.024 | -0.0465 | No |
| 19 | ALDH3B2 | na | 13954 | 0.019 | -0.0595 | No |
| 20 | ENO1 | na | 14982 | 0.011 | -0.0871 | No |
| 21 | PDHB | na | 15297 | 0.009 | -0.0945 | No |
| 22 | PFKM | na | 15872 | 0.005 | -0.1102 | No |
| 23 | ACSS2 | na | 16411 | 0.001 | -0.1255 | No |
| 24 | ADH1B | na | 16426 | 0.001 | -0.1258 | No |
| 25 | GAPDH | na | 16727 | -0.001 | -0.1342 | No |
| 26 | TPI1 | na | 17233 | -0.005 | -0.1478 | No |
| 27 | PDHA1 | na | 17379 | -0.006 | -0.1509 | No |
| 28 | PGM2 | na | 17963 | -0.010 | -0.1658 | No |
| 29 | BPGM | na | 18095 | -0.011 | -0.1676 | No |
| 30 | PKM | na | 18421 | -0.013 | -0.1745 | No |
| 31 | ADH7 | na | 18569 | -0.014 | -0.1761 | No |
| 32 | PFKL | na | 19009 | -0.017 | -0.1856 | No |
| 33 | ALDOC | na | 19211 | -0.018 | -0.1880 | No |
| 34 | HK1 | na | 19947 | -0.023 | -0.2049 | No |
| 35 | PGAM4 | na | 20018 | -0.024 | -0.2025 | No |
| 36 | ALDH3A1 | na | 20262 | -0.026 | -0.2047 | No |
| 37 | PKLR | na | 21593 | -0.036 | -0.2364 | No |
| 38 | ALDOA | na | 21826 | -0.038 | -0.2361 | No |
| 39 | ALDOB | na | 22525 | -0.045 | -0.2478 | Yes |
| 40 | LDHB | na | 22665 | -0.047 | -0.2431 | Yes |
| 41 | PGAM1 | na | 22679 | -0.047 | -0.2348 | Yes |
| 42 | DLAT | na | 22996 | -0.050 | -0.2346 | Yes |
| 43 | GPI | na | 23113 | -0.051 | -0.2284 | Yes |
| 44 | PGK1 | na | 23308 | -0.054 | -0.2240 | Yes |
| 45 | ADH5 | na | 23842 | -0.060 | -0.2282 | Yes |
| 46 | LDHA | na | 24699 | -0.072 | -0.2395 | Yes |
| 47 | PGM1 | na | 25044 | -0.077 | -0.2352 | Yes |
| 48 | ALDH1B1 | na | 25126 | -0.078 | -0.2231 | Yes |
| 49 | ALDH9A1 | na | 25157 | -0.078 | -0.2095 | Yes |
| 50 | GCK | na | 25358 | -0.081 | -0.2002 | Yes |
| 51 | G6PC2 | na | 25991 | -0.090 | -0.2017 | Yes |
| 52 | HK2 | na | 26843 | -0.105 | -0.2067 | Yes |
| 53 | ALDH1A3 | na | 28596 | -0.146 | -0.2301 | Yes |
| 54 | PGAM2 | na | 30269 | -0.197 | -0.2418 | Yes |
| 55 | PCK2 | na | 30472 | -0.203 | -0.2099 | Yes |
| 56 | LDHAL6B | na | 31068 | -0.223 | -0.1857 | Yes |
| 57 | ALDH2 | na | 31150 | -0.226 | -0.1460 | Yes |
| 58 | GALM | na | 31318 | -0.232 | -0.1076 | Yes |
| 59 | ADH1C | na | 33242 | -0.340 | -0.0999 | Yes |
| 60 | ADH1A | na | 33507 | -0.360 | -0.0406 | Yes |
| 61 | PGK2 | na | 33918 | -0.408 | 0.0234 | Yes |
Table: GSEA details [plain text format]

  

Fig 2: KEGG\_GLYCOLYSIS\_GLUCONEOGENESIS      
 Blue-Pink O' Gram in the Space of the Analyzed GeneSet

  

Fig 3: KEGG\_GLYCOLYSIS\_GLUCONEOGENESIS: Random ES distribution      
 Gene set null distribution of ES for **KEGG\_GLYCOLYSIS\_GLUCONEOGENESIS**
